# Smoke or smokeless moxibustion treatment for breech presentation: A three‐arm pilot trial

**DOI:** 10.1111/jjns.12426

**Published:** 2021-05-17

**Authors:** Akiko Higashihara, Shigeko Horiuchi

**Affiliations:** ^1^ Department of Nursing Saitama Prefectural University Saitama Japan; ^2^ Graduate School of Nursing Science St. Luke's International University Tokyo Japan

**Keywords:** breech presentation, moxibustion, pilot trial, quasi‐experimental study

## Abstract

**Aims:**

We conducted a pilot trial to compare the effects of smoke and smokeless moxibustion with a control as a possible supplement to external cephalic version (ECV) for converting breech to cephalic presentation and increasing adherence to cephalic position, and to assess their effects on the well‐being of the mother and child.

**Methods:**

We used a quasi‐experimental design with 3 arms: a smoke moxibustion (SM) (*n* = 20) and smokeless moxibustion (SLM) (*n* = 20) groups (20‐min acupoint BL67 stimulation once or twice daily for 10–14 days), and a control group (*n* = 20). The participants had singleton breech presentations between 33 and 35 gestation weeks. The primary outcome was cephalic presentation at the conclusion of intervention. The secondary outcomes were cephalic presentation at birth and effects on mother and child well‐being.

**Results:**

At the conclusion of intervention, cephalic presentation was higher in the SLM (60.0%) than the control groups (25.0%), Relative Risk 2.40, 95% Confidence Interval [1.04–5.56]; there was no significant difference for SM. At birth, there were no significant differences in cephalic presentation or well‐being.

**Conclusion:**

SLM treatment showed an increasing trend towards cephalic presentation at the conclusion of intervention. Although significant differences were not observed at birth possibly due to the small samples and non‐randomization, moxibustion was safe, and not associated with perinatal morbidity and mortality. A randomized controlled trial with a larger sample is warranted to ascertain SLM treatment as a possible ECV supplement for converting and increasing adherence to cephalic position.

## 1 INTRODUCTION

The breech presentation is a major condition for performing elective cesarean section. Singh et al. (2020) previously described the various indications for cesarean section. They reported that the primary, secondary, and tertiary indications for elective cesarean section were previous cesarean section (33%), fetal distress (19%), and malpresentations (13%), respectively. Breech presentation is reportedly the main indication for elective cesarean section. Thus, to increase spontaneous fetal movement towards cephalic presentation, the development of methods for correcting breech presentation in pregnant women who desire to have a vaginal cephalic birth is needed. Methods of effective care based on evidence for converting breech presentation to cephalic presentation must also be carefully examined.

The proportion of breech presentation is usually approximately 20% at 28 weeks of gestation (Cunningham et al., 2014, pp. 558–573; Westgren et al., 1985). However, the majority subsequently convert into cephalic presentation, decreasing the proportion to 3–4% of the fetuses at full term (Cunningham et al., 2014, pp. 558–573; Hickok et al., 1992). Some of the methods for converting to cephalic presentation include posture management, moxibustion alone or together with acupuncture, and external cephalic version (ECV). When planning to correct the fetal position, the timing of physical manipulative intervention is highly crucial. For example, the critical timing for physical manipulative interventions such as ECV is before or after 37 weeks of gestation, which is the border between preterm birth and full‐term birth. However, this is not an issue for non‐manipulative interventions such as moxibustion even before 37 weeks of gestation. The effects of posture management (e.g., the knee‐to‐chest position) for correcting fetal presentation still need further clarification (Hofmeyr & Kulier, 2012). ECV at term is reported to decrease non‐cephalic presentation at delivery, Relative Risk (RR) 0.42, 95% Confidence Interval (CI) [0.29, 0.61], and cesarean section, RR 0.57, 95% CI [0.40, 0.82] (Hofmeyr et al., 2015). However, Hutton et al. (2015) showed evidence that the risk of preterm labor was increased with early ECV compared with ECV after 37 weeks, RR 1.51, 95% CI [1.03, 2.21]. Therefore, the timing of the implementation of ECV should be carefully considered.

Moxibustion is a traditional Chinese medicine that offers an alternative approach for inducing cephalic version of breech presentation. Moxibustion generates heat by burning a herbal preparation containing *Artemisia vulgaris* (mug‐wort) in the moxibustion stick, which is placed near the acupuncture point to induce a warming sensation (Turner & Low, 1987). To promote cephalic version, the acupuncture point bladder 67 (BL67; located on the outer corner of the fifth toenail) is stimulated using a moxibustion stick (Cardini et al., 1991). Studies have shown that pregnant women have significantly more subjective fetal movements during moxibustion (Cardini & Weixin, 1998; Guittier et al., 2009). This suggests that fetal movements may be increased and that fetal self‐rotation may be promoted. The specific mechanism of the action has yet to be determined.

Three systematic reviews have reported the effectiveness and safety of moxibustion for breech presentation (Coyle et al., 2012; Vas et al., 2009; Zhang et al., 2013). Meta‐analyses have also been performed to compare moxibustion usage with no treatment. When moxibustion was combined with acupuncture and compared with no treatment, the moxibustion‐acupuncture combination resulted in fewer non‐cephalic presentations at birth, RR 0.73, 95% CI [0.57, 0.94], 1 trial, 226 women, and fewer births by cesarean section, RR 0.79, 95% CI [0.64, 0.98] (Coyle et al., 2012). Moreover, a higher rate of cephalic version was found in the moxibustion group than in the control group, RR 1.36, 95% CI [1.17, 1.58] (Vas et al., 2009). However, significant heterogeneity was evident among the systematic reviews. In these previous systematic reviews, there were trials for different moxibustion methods. Particularly for the types of moxibustion, the trials using smoke moxibustion and smokeless moxibustion were mixed. Thus, a unified study of moxibustion methods (i.e., homogenous methodology) is thought to be necessary.

To the best of our knowledge, there are presently no studies comparing and verifying smoke moxibustion, smokeless moxibustion, and control treatments for breech presentation as a three‐arm pilot trial. Moreover, regarding smoke moxibustion versus smokeless moxibustion, it remains unclear which is more effective, safe, and acceptable to pregnant women, and whether there is any difference in the amount of heat generated. Several studies have shown the effects of moxibustion smoke. Coulon et al. (2014) reported that out of 164 patients in the moxibustion group (with acupuncture), two patients complained of nausea or vomiting. In Cardini et al.'s (2005) randomized controlled trial (RCT) of Italians, 14 out of 65 patients in the intervention group had events such as nausea and throat problems due to smoke moxibustion. As the moxibustion used in the above two RCTs was smoke moxibustion, it is reported that its use may cause nausea and throat problems. Therefore, to successfully establish an effective moxibustion method for breech presentation, a pilot study is necessary to compare smoke moxibustion and smokeless moxibustion simultaneously. Thus, this research was conducted as a pilot study involving a non‐randomized controlled trial as a preliminary step towards a future randomized controlled trial.

The specific aims of this pilot study were (a) to compare the effects of smoke moxibustion and smokeless moxibustion treatments with the control group as a possible supplement to ECV for converting breech presentation to cephalic presentation and increasing adherence to the newly obtained cephalic position, and (b) to assess the effects of these treatments on the well‐being of the mother and child.

In line with these aims, we suggest and examine three hypotheses.

Hypothesis 1 The smoke moxibustion stick group (SM group) and smokeless moxibustion stick group (SLM group) will have higher rates of cephalic presentation after treatment than the control group.

Hypothesis 2 The SM group and SLM group will have higher rates of cephalic presentation at birth than the control group.

Hypothesis 3 There will be no significant differences in the well‐being of the mother and child among the three groups in terms of the following outcomes: premature birth, premature rupture of membranes (PROM) at <37 weeks, Apgar score <7 at 5 min, umbilical cord blood pH <7.1, admission to neonatal intensive care unit (NICU), and intrauterine fetal death.

## 2 METHODS

### 2.1 Study design

We used a quasi‐experimental design with three arms for this pilot trial.

### 2.2 Participants and setting

Eligible participants were allocated into the following groups: (a) SM group, (b) SLM group, and (c) control group. This study was conducted in two perinatal medical centers, a maternity hospital, and an obstetrics and gynecology clinic in Tokyo, Japan, between March 2016 and January 2017.

#### 2.2.1 Inclusion criteria

The inclusion criteria were as follows:

1. Pregnant women with singleton breech presentations between 33 and 35 gestation weeks diagnosed by physical examination and ultrasound.
2. Japanese women aged 18 years and above, with normal fetal biometry, and with normal progression of pregnancy.

#### 2.2.2 Exclusion criteria

The exclusion criteria were as follows:

1. Non‐obstetric complications: Maternal heart or kidney disease.
2. Obstetric complications: Pregnancy with multiples of twins and beyond, risk of preterm birth (preterm uterine contractions, initial dilatation, or shortening of the cervix with a score of 4 on the Bishop scale; tocolytic therapy), uterine fibroids >4 cm, placenta previa, hypertensive disorders of pregnancy, PROM.
3. Contraindication to vaginal delivery: Previous uterine surgery, uterine malformations, bone pelvic defects.
4. Fetal conditions: Intrauterine growth restriction, fetal malformation, or chromosomal disorder.
5. Conditions to avoid in interventions: Pregnant woman or siblings of the fetus diagnosed with bronchial asthma or a pulmonary problem and are treated; allergies to *Artemisia vulgaris*; pregnant woman or siblings of the fetus who have symptoms of coughing, respiratory discomfort from smoke, and prior moxibustion treatment to achieve fetal version.

#### 2.2.3 Sample size

This three‐arm pilot study was conducted in preparation for a future RCT. The sample size was estimated based on a previous feasibility study which involved a sample of 30 women and reported on moxibustion for cephalic version (Do et al., 2011). In this previous feasibility study which was a two‐arm trial, 20 women were eventually allocated and analyzed. In our present pilot study, we compared three groups and enrolled 60 women (i.e., 20 women in each group) with consideration of those lost to follow‐up.

#### 2.2.4 Allocation of participants

First, participants were recruited for the SM group until the required number of 20 people was secured. Second, participants were recruited for the SLM group until the required number of 20 people was secured. Third, participants were recruited for the control group until the required number of 20 people was secured. At the end of the recruitment for the first group, posters in the facilities requesting participation were replaced with other posters designed to recruit the next group. The pilot trial was stopped when enrollment of the required number of women and performance of the intervention treatments were completed.

### 2.3 Interventions

#### 2.3.1 Smoke moxibustion stick method

The SM method is a traditional moxibustion treatment which is presently the conventional smoke moxibustion stick method used. Participants in the SM group self‐administered the moxibustion treatment at home. The acupuncture point BL67 (*Zhiyin* in Chinese), which is located close to the outer angle of the little toenail, was stimulated using a smoke moxibustion stick for 20 min once or twice daily for 10–14 days. The women performed the moxibustion treatment in relaxed clothes and posture. It was recommended that they sit down on a sofa or lean against a wall with cushions, and assume a comfortable posture. The heated moxibustion stick was placed on a stand especially made for the stick. The heated moxibustion stick was then applied for 10 min on each foot for a total of 20 min per treatment at BL67. The heated tip of the moxibustion stick was applied from a distance of 1.5–3 cm. The participants performed the treatment once or twice daily for 10–14 days, recording their self‐administered treatment in a moxibustion diary. The moxibustion diary and physical condition check sheet were provided to the participants. For the physical condition at pre‐treatment and post‐treatment, the participants checked for the presence or absence of uterine contraction, vaginal bleeding, and membrane rupture using the physical condition check sheet. The items in this check sheet, as well as the timeline and treatment protocol, were based on systematic reviews of possible adverse events and moxibustion for breech presentation (Coyle et al., 2012; Vas et al., 2009; Zhang et al., 2013). The participants were given a moxibustion set that can be used for 14 days. Each set contained the following: moxibustion sticks, moxibustion stick stand, ashtray, lighter, fire extinguishing tool, trays, deodorizing spray, aluminum seats, and cap for safekeeping the moxibustion sticks. Follow‐up was made by phone on day 1 and after 7 days to confirm the application and safety of the moxibustion treatment, and check on whether there were any questions or concerns.

#### 2.3.2 Smokeless moxibustion stick method

The SLM method uses a stick that is odorless and carbonized, and the smoke can be maximally controlled. Participants in the SLM group self‐administered the moxibustion treatment at home. BL67 was stimulated using a smokeless moxibustion stick for 20 min once or twice daily for 10–14 days. This procedure was identical to the smoke moxibustion technique except for the use of a stick that is carbonized with the smoke capable of being maximally controlled.

#### 2.3.3 Control group

Participants in the control group received leaflets explaining about (a) adequate sleep, balanced diet, exercise, rest, and stress‐reduction that they need to pay attention to in their daily life, (b) important aspects of uterine contraction, and (c) lying down positions that they have to assume depending on the location of the fetal spine. In Japan, obstetricians perform an ultrasound almost every time during prenatal check‐ups. Particularly in the third trimester of pregnancy, when the baby is in the breech presentation, an ultrasound is performed every time to inform and educate the pregnant woman regarding the fetal position. Thus, the pregnant woman knows the position of the fetal spine. The participants were also instructed not to have any moxibustion treatment but to spend their time naturally until the next medical check‐up after 10–14 days.

### 2.4 Outcome measures

The primary outcome was the rate of cephalic presentation after 10–14 days from commencement of the intervention. Fetal presentation was determined by ultrasound. The secondary outcomes were the rate of cephalic presentation at birth, mode of birth, maternal outcomes (number of ECV), and well‐being of the mother and child (related to perinatal morbidity and mortality: premature birth, PROM at <37 weeks, Apgar score <7 at 5 min, umbilical cord blood pH < 7.1, admission to NICU, and intrauterine fetal death).

#### 2.4.1 Independent measures

The following information was obtained at baseline: (a) demographic data: age, educational level, (b) obstetric‐gynecological variables: parity, gestational age at the start of treatment, height (centimeters [cm]), weight (kilograms), body mass index (BMI), employment status, sensitivity to cold (a risk factor that can precipitate premature birth, PROM, weak labor pains, prolonged labor, and atonic bleeding) (Nakamura & Horiuchi, 2013), and (c) factors related to breech presentation: gestational age at the start of breech presentation, placental location, estimated fetus weight at the start of treatment (grams), amniotic fluid depth (cm), type of presentation (breech or transverse), umbilical cord length at birth (cm), and coiling of the umbilical cord at the start of treatment. These data were collected using the clinical records and a self‐report questionnaire. The participants completed the self‐report questionnaire before and after the intervention.

### 2.5 Procedures

This study used a quasi‐experimental design. Three arms were allocated as follows: (a) SM group (*n* = 20), (b) SLM group (*n* = 20), and (c) control group (*n* = 20). For moxibustion administration, the participants self‐administered the moxibustion treatment at their home. We adopted this procedure based on the method of Cardini and Weixin (1998) who reported the effectiveness of self‐administered moxibustion for breech presentation by the participants at home. The treatment protocol was supervised by Dr. S.K., an acupuncture and moxibustion specialist for pregnant women with 35 years of clinical experience.

Regarding the recruitment setting, cooperation with research facilities was sought using a “request document to use a facility.” If approval of the sought cooperation was obtained, this was posted in the recruitment section of the bulletin board of the outpatient ward as a form of information disclosure of this research. Recruitment posters were put up on the bulletin boards for about 3 to 4 months per group, for a total of 11 months during the study period.

The first author (A.H.) and research assistant (RA) midwives who acted as the intervention staff were directly involved in this research. The intervention staff previously underwent the same training on how to perform the moxibustion method. The intervention staff clearly explained the purpose of the research, adequately described the contents of the intervention and control groups, and efficiently administered the questionnaire. The intervention staff performed the same work, carried out recruitment, explained the research contents to pregnant women, and assisted in data collection. A video providing clear instructions on the moxibustion method was also created and provided to ensure that the same instructions and guidance would be given to all participants. After the 31–33 gestational weeks medical check‐up of pregnant women, those who met the eligibility criteria were identified from the reservation list for the next regular prenatal medical check‐up. At the 33–35 gestation weeks medical check‐up, women who were diagnosed with breech presentation by ultrasound were sampled conveniently by an obstetrician and gynecologist specialist, and were requested to participate in the present study, with the intervention staff explaining that it would take 30 min to introduce the research after the prenatal check‐up. For women who indicated interest, the intervention staff carefully explained the procedure in a private room, creating an atmosphere where it was easy to ask questions. Once consent to participate in the research was obtained, each participant received and completed a pre‐intervention self‐report questionnaire. The post‐intervention questionnaire was given to each participant at the next antenatal check‐up after the intervention. The intervention group participants were provided advice and training using instruction documents on the safe use the moxibustion stick including fire safety education, and the intervention procedure as demonstrated by the intervention staff. The pregnant women were checked by the intervention staff as to whether they were able to acquire the skills required to properly self‐administer moxibustion. In addition, the intervention group participants were provided access information for the video instructional website for the moxibustion method at home so that they could watch the video every time and check the precautions. The teaching materials were prepared under the supervision of a qualified practitioner of acupuncture and moxibustion. Some possible side effects in the intervention group were (a) burns, (b) feelings of unpleasantness, and (c) uterine contraction, PROM, or bleeding as induced by moxibustion. The possible adverse events and corresponding first aid were clearly explained and written in the explanation documents. The phone number of the researcher (A.H.) was also written in the explanation documents for any urgent requests for consultation regarding adverse events. For the control group, the intervention staff advised the participants not to undergo any acupuncture, moxibustion, or massage treatment to correct the breech presentation during the study period.

Ultrasound was performed to check the fetal position in all the participants of the three groups at 10 to 14 days after the intervention. If the fetus was still in breech presentation, the participants could choose the ECV treatment if needed.

### 2.6 Ethical considerations

The Institutional Review Board of St. Luke's International University, Tokyo, Japan approved this study (No. 15–086). This study was registered in the Clinical Trials Registry of University Hospital Medical Information Network in Japan after approval by the Ethics Screening Committee (UMIN000021377). All participants provided written informed consent.

### 2.7 Data analysis

Statistical analyses were conducted for all data. To evaluate the differences among the groups, continuous variables were analyzed using Student's *t* test and one‐way analysis of variance (ANOVA). Categorical variables were analyzed using the Fisher exact test or Chi‐square test. The relative risk (RR) and 95% CI were calculated for the SM versus the control groups and for the SLM versus the control groups. All analyses were performed using International Business Machines Corporation (IBM) Statistical Package for Social Science (SPSS version 22.0, IBM Japan, Tokyo, Japan). The level of statistical significance was set at *p* < 0.05.

## 3 RESULTS

**FIGURE 1:**
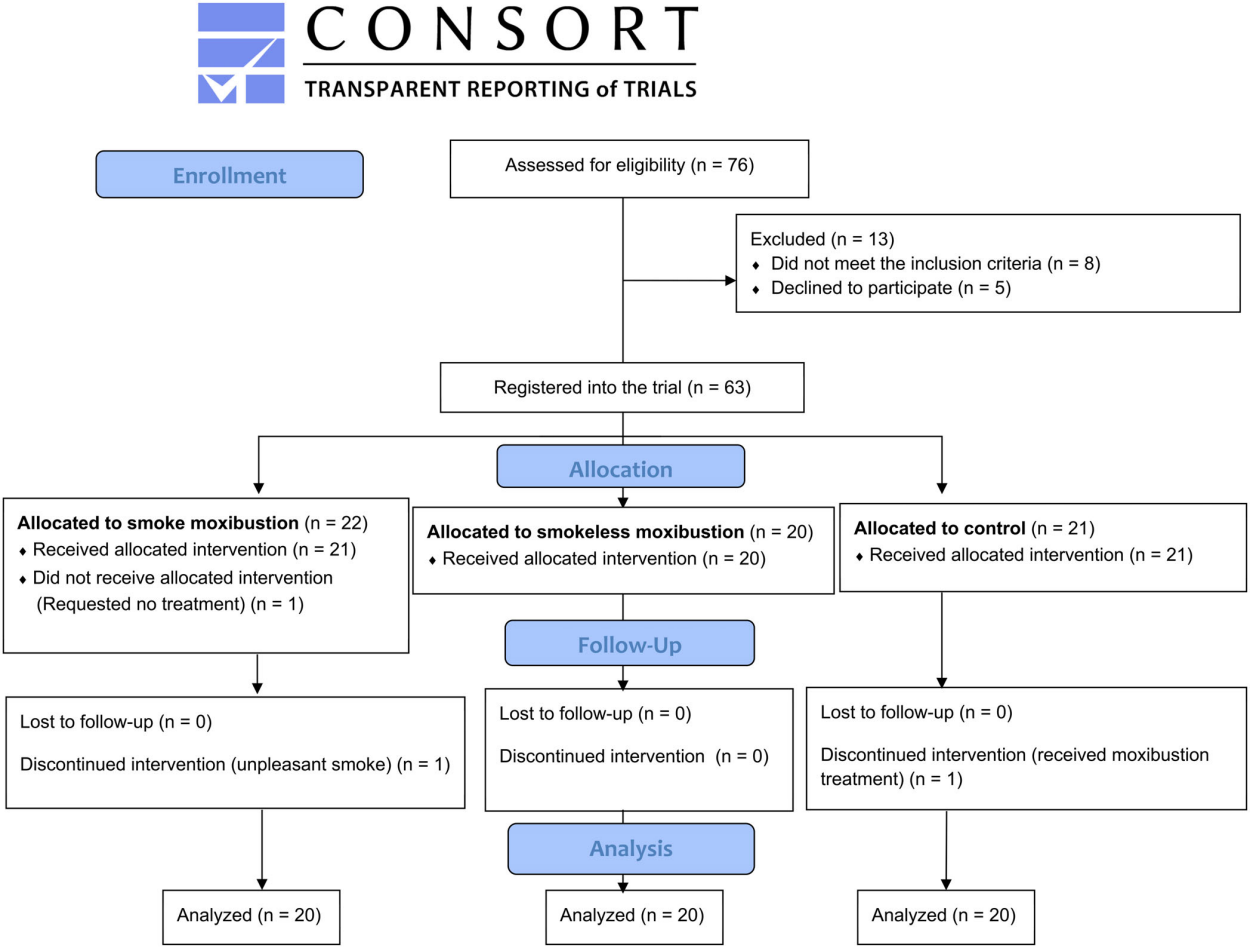
Participant flow diagram

The participants were recruited and followed up from March 2016 to January 2017. A total of 63 women were registered in this trial. In the end, there were 20 participants in each group for analysis. The participant flow diagram is shown in Figure 1.

### 3.1 Characteristics of the study participants

There were no significant differences between groups in the sociodemographic characteristics, obstetric‐gynecological variables, and related factors of breech presentation as baseline characteristics of the study participants (Table 1).

**TABLE 1:** Baseline characteristics of the study participants

	Smoke moxibustion	Smokeless moxibustion	Control	*p*‐Value
	(*n* = 20)	(*n* = 20)	(*n* = 20)	
Age (years): mean (SD)	33.9 (4.64)	32.9 (5.12)	34.8 (3.85)	0.426
Education: *n* (%)				
Junior high or high school	2 (10.0)	4 (20.0)	0 (0.0)	0.196
Junior college	4 (20.0)	8 (40.0)	5 (25.0)	
Degree	11 (55.0)	6 (30.0)	13 (65.0)	
Graduate	3 (15.0)	2 (10.0)	2 (10.0)	
Gestational age at start of treatment (weeks): mean (SD)	34.0 (0.78)	34.1 (0.80)	34.3 (0.61)	0.307
Parity: *n* (%)				
Primipara	14 (70.0)	12 (60.0)	14 (70.0)	0.741
Multipara	6 (30.0)	8 (40.0)	6 (30.0)	
Maternal height (cm): mean (SD)	160.3 (5.63)	159.7 (5.59)	160.3 (4.97)	0.927
Maternal weight (kg): mean (SD)	52.8 (5.47)	52.0 (7.64)	51.6 (9.61)	0.889
BMI: mean (SD)	20.5 (1.59)	20.4 (2.51)	20.1 (3.79)	0.902
Working: *n* (%)	15 (75.0)	11 (55.0)	14 (70.0)	0.481
Sensitivity to cold: *n* (%)	17 (85.0)	15 (75.0)	16 (80.0)	0.918
Gestational age at start of breech (weeks): mean (SD)	29.1 (3.22)	29.7 (3.94)	28.2 (2.51)	0.357
Placenta location: *n* (%)				
Anterior	11 (55.0)	8 (40.0)	5 (26.3)^a^	0.133
Posterior	4 (20.0)	8 (40.0)	8 (42.1)	
Side	0 (0.0)	1 (5.0)	4 (21.1)	
Fundus	5 (25.0)	3 (15.0)	2 (10.5)	
Estimated fetus weight (g): mean (SD)	2065.8 (202.32)	2169.2 (241.85)	2167.7 (204.66)	0.234
Amniotic fluid depth (cm): mean (SD)	3.7 (0.93)	3.7 (0.71)	3.7 (0.87)	0.998
Type of presentation: *n* (%)				
Breech	18 (90.0)	19 (95.0)	19 (95.0)	1.00
Transverse	2 (10.0)	1 (5.0)	1 (5.0)	
Umbilical cord length (cm): mean (SD)	56.9 (13.97)	55.1 (12.83)	51.55 (6.68)	0.347
Coiling of umbilical cord: *n* (%)	8 (40.0)	7 (35.0)	5 (25.0)^a^	0.738

*Note: p*‐Value: One‐way analysis of variance or Fisher's exact test for comparisons between groups.

Abbreviations: BMI, body mass index; SD, standard deviation.

^a^ Missing data n = 1.

### 3.2 Primary outcome

#### 3.2.1 Cephalic presentation at intervention conclusion (after 10–14 days)

Nine fetuses in the SM group (45%), 12 fetuses in the SLM group (60%), and 5 fetuses in the control group (25%) converted to cephalic presentation at the conclusion of intervention. The proportion of cephalic version was higher in the SLM group (60%) than in the control group (25%), RR 2.40, 95% CI [1.04, 5.56]. There was no significant difference between the SM group and the control group, RR 1.80, 95% CI [0.73, 4.43] (Table 2).

**TABLE 2:** Comparison of outcomes between moxibustion and control groups

Outcomes	Smoke moxibustion	Smokeless moxibustion	Control	RR [95% CI]	RR [95% CI]
	(*n* = 20)	(*n* = 20)	(*n* = 20)	Smoke moxibustion vs control	Smokeless moxibustion vs control
Cephalic presentation at the intervention conclusion	9/20 (45.0)	12/20 (60.0)	5/20 (25.0)	1.80 [0.73, 4.43]	2.40 [1.04, 5.56]
Spontaneous cephalic version after intervention: *n*	1	0	1		
Successful ECV: *n*/underwent ECV: *n*	0/3	0/0	3/5		
Cephalic presentation at birth					
Including ECV/including ECV	10/20 (50.0)	12/20 (60.0)	9/20 (45.0)	1.11 [0.58, 2.14]	1.33 [0.73, 2.44]
Excluding ECV/including ECV	10/20 (50.0)	12/20 (60.0)	6/20 (30.0)	1.67 [0.75, 3.71]	2.00 [0.94, 4.27]
Excluding ECV/excluding ECV	10/17 (58.8)	12/20 (60.0)	6/15 (40.0)	1.47 [0.70, 3.07]	1.50 [0.73, 3.07]
Cesarean section					
Including ECV/including ECV	10/20 (50.0)	10/20 (50.0)	13/20 (65.0)	0.77 [0.45, 1.33]	0.77 [0.45, 1.33]
Excluding ECV/excluding ECV	7/17 (41.2)	10/20 (50.0)	9/15 (60.0)	0.69 [0.34, 1.39]	0.83 [0.46, 1.52]

*Note*: Data are expressed as *n* (%).

Abbreviations: CI, Confidence Interval; ECV, External Cephalic Version; RR, Relative Risk.

The protocol of this study was to perform moxibustion for 20 min once or twice daily for 10–14 days. The mean number of times of performing moxibustion was 17.65 times (SD 4.30, range 10–26 times) in the SM group and 19.95 times (SD 5.54, range 7–28 times) in the SLM group. The mean number of days of implementation was 12.65 days (SD 1.53, range 10–14 days) in the SM group and 12.65 days (SD 1.87, range 7–14 days) in the SLM group. There was no significant difference between the groups.

These results showed that the rate of cephalic presentation after 10–14 days of the intervention was higher in the SLM group than in the control group. This indicates that Hypothesis 1 is supported for the SLM group, but not for the SM group.

### 3.3 Secondary outcomes

#### 3.3.1 Cephalic presentation at birth

In the calculation of the proportion of cephalic presentation at birth, the practice of ECV is considered a factor that affects the fetal position at birth. To examine the effects of moxibustion, we conducted analysis in ways which both included ECV practitioners, and also excluded ECV practitioners, in comparisons among the three groups.

First, numbers which included the women who underwent ECV were used for both the numerators and denominators in calculating the proportion of cephalic presentation. As a result, there was no significant difference between the intervention groups and the control group.

Next, only numbers which excluded women who underwent ECV were used for the numerators in making the same calculations. As a result, the gap increased between the proportion of cephalic version in the SLM group (60%) versus the control group (30%), RR 2.00, 95% CI [0.94, 4.27]. However, this difference between the two groups still did not reach a level of significance.

Third, numbers which excluded women who underwent ECV were used for both the numerators and denominator for the calculations. As a result, there were again no significant differences between either intervention group and the control group (Table 2).

Thus, Hypothesis 2 was not supported by the present results.

Regarding cesarean sections, we again analyzed two patterns: including those who underwent ECV and excluding those who underwent ECV. For calculations including those who underwent ECV, 10 women each in the SM and SLM groups had cesarean sections compared with 13 women in the control group, yielding the same values for each, RR 0.77, 95% CI [0.45, 1.33]. For calculations excluding those who underwent ECV, 7 of 17 women in the SM group, and 10 of 20 women in the SLM group had cesarean sections, compared with 9 of 15 women in the control group, yielding RR 0.69, 95% CI [0.34, 1.39] for the SM group, and RR 0.83, 95% CI [0.46, 1.52] for the SLM group. Overall, there were no significant differences in the proportions of cesarean section among the three groups.

### 3.4 Comparison of the effects of the intervention treatments on the well‐being of the mother and child among groups

There were no significant differences in the well‐being of the mother and child in all the outcomes (Table 3). Thus, Hypothesis 3 was supported by the present results.

**TABLE 3:** Comparison of the effects of treatment intervention on the well‐being of the mother and child among groups

	Smoke moxibustion	Smokeless moxibustion	Control	*p*‐Value
	(*n* = 20)	(*n* = 20)	(*n* = 20)	
Premature birth: *n* (%)	0 (0.0)	0 (0.0)	0 (0.0)	N/A
Premature rupture of membranes at <37 weeks: *n* (%)	0 (0.0)	0 (0.0)	0 (0.0)	N/A
Gestational age at birth: mean (SD)	39.3 (1.14)	39.4 (1.15)	39.1 (0.93)	0.542
Apgar score < 7 at 5 min: n (%)	1 (5.0)	0 (0.0)	0 (0.0)	N/A
Umbilical cord blood pH < 7.1: n (%)	0 (0.0)	0 (0.0)	0 (0.0)	N/A
Birth weight (g): mean (SD)	2,994.3 (414.36)	3,188.9 (448.85)	2,987.5 (311.73)	0.198
Admission to NICU: *n* (%)	1 (5.0)	0 (0.0)	0 (0.0)	N/A
Intrauterine fetal death: *n* (%)	0 (0.0)	0 (0.0)	0 (0.0)	N/A

Abbreviations: NICU, neonatal intensive care unit; SD, standard deviation.

## 4 DISCUSSION

### 4.1 Primary outcome

Our results showed that at the conclusion of intervention, the proportion of cephalic version was significantly higher in the SLM group than in the control group. However, no significant difference was observed between the SM group and the control group. Two RCTs have shown different conclusions on the use of smokeless moxibustion sticks as reported by Do et al. (2011) and Guittier et al. (2009). Do et al. (2011) found a trend towards an increase in cephalic version at the conclusion of the intervention for women receiving moxibustion compared with the control. The treatment protocol involved 20 applications of heat stimulation using smokeless moxibustion (i.e., 20 min twice daily for 10 days). Guittier et al. (2009) found no difference in the proportion of versions among women treated with moxibustion and women in the control group. In the protocol of Guittier et al. (2009), the maximum number of moxibustion sessions was 14 times. In our present study protocol, the average number of moxibustion treatments was 19.95 times, which showed the effects of moxibustion. Therefore, we consider our protocol to be more effective. Based on these results, it is therefore important to identify the optimal number of times of rigorous moxibustion application. We found that heat stimulation using moxibustion is necessary for at least 20 times between 10 and 14 days, and this should to be incorporated into the protocol.

Some studies showed the effectiveness of smoke moxibustion for breech presentation, whereas other studies did not. In their study of 260 Chinese pregnant women, Cardini and Weixin (1998) showed a higher cephalic conversion ratio in the smoke moxibustion group than in the control group, RR 1.58, 95% CI [1.29, 1.94]. Cardini et al. (2005) found no significant difference, however, in the cephalic conversion ratio between the smoke moxibustion group and the control group in 123 Italian pregnant women, RR 0.95, 95% CI [0.59, 1.5]. Bue and Lauszus (2016) reported similar findings in 200 Danish women, RR 1.05, 95% CI [0.8, 1.38].

Overall, it appears that previous studies have significant differences and large sample sizes. Thus, in future studies, changes in temperature from the feet to the abdomen should be measured by thermography and included as data. Additionally, improvement of the intervention protocol using various strengths of moxibustion heat should be carefully considered and incorporated.

### 4.2 Secondary outcomes

Women who performed smoke and smokeless moxibustion stick treatments showed an increasing trend in cephalic version at birth compared with women in the control group. During birth, however, there were no significant differences among the three groups. As ECV was considered to be a factor that has an effect on the outcome, analysis was also conducted with women who underwent ECV excluded. The exclusion of ECV patients reduced the overall number of eligible women, therefore, the small sample size of the pilot study this did not result in any significant differences between the groups.

Notably, some previous studies have included women who underwent ECV in the analysis (Bue & Lauszus, 2016; Cardini et al., 2005; Cardini & Weixin, 1998; Guittier et al., 2009; Vas et al., 2013). Some of these studies (Cardini & Weixin, 1998; Vas et al., 2013) showed that moxibustion at BL67 is effective in correcting breech presentation whereas others revealed the opposite effect (Bue & Lauszus, 2016; Cardini et al., 2005; Guittier et al., 2009). These results imply that it is necessary to have a larger sample size that will have a sufficient effect even if the women who undergo ECV are included. Thus, it is necessary to calculate the sample size to be able to reliably verify the effects of moxibustion, and to increase the number of subjects in future trials.

### 4.3 Effects of the treatment intervention on the well‐being of the mother and child among groups

We found no significant difference in the well‐being of the mother and child (i.e., related to perinatal morbidity and mortality: premature birth, PROM at <37 weeks, Apgar score <7 at 5 min, umbilical cord blood pH < 7.1, admission to NICU, and intrauterine fetal death) among the three groups. Previous systematic reviews showed no significant difference in preterm birth between the moxibustion group, RR 0.95, 95% CI [0.23, 3.92] (Vas et al., 2009) and the control group, RR 0.92, 95% CI [0.35, 2.47] (Zhang et al., 2013). There was also no significant difference in PROM, RR 0.82, 95% CI [0.007, 9.31] (Coyle et al., 2012); RR 0.54, 95% CI [0.10, 3.08] (Vas et al., 2009); RR 1.55, 95% CI [0.17, 14.35] (Zhang et al., 2013). The results of the present study are similar to the above‐mentioned results of the previous systematic reviews.

Overall, the results showed that moxibustion was not harmful to the pregnant woman and fetus in the third trimester of pregnancy, as well as to the newborn baby. We therefore consider moxibustion as one of the effective complementary alternative medicines for pregnant women.

### 4.4 Study limitations and future research

In the present study, selection bias may not have been completely excluded because of the lack of random allocation. Also, the sample size was small and the setting was limited to four tertiary obstetric hospitals in Tokyo.

In future studies, smokeless moxibustion will be used in the treatment protocol and moxibustion treatment will be performed 20 times between 10 and 14 days as in the protocol of this pilot study. RCT with a larger sample size will be needed to verify the effects of moxibustion even if women with ECV are included. It is also necessary to investigate whether moxibustion can be a secondary treatment to ECV, wherein moxibustion before ECV can promote conversion into a more cephalic version.

## 5 CONCLUSION

Women who performed SLM treatment for 20 min once or twice daily for 10–14 days showed an increasing trend towards cephalic presentation at the conclusion of intervention compared with women in the control group. This was not evident for the SM treatment. Although significant differences in cephalic presentation at birth and effects on well‐being of the mother and child were not observed at birth, possibly due to the small sample size and non‐randomization, moxibustion was safe, and not associated with perinatal morbidity and mortality. With the potential of SLM treatment to increase cephalic position, a future RCT with a larger sample size should be explored to ascertain SLM treatment as a possible supplement to ECV for converting breech presentation to cephalic presentation and increasing adherence to the newly obtained cephalic position.

## CONFLICT OF INTERESTS

The authors declare that they have no competing interests associated with this study. There are no conflicts of interests for this study.

## AUTHORS' CONTRIBUTIONS

Akiko Higashihara designed the study, collected and analyzed the data, and drafted the initial manuscript. Shigeko Horiuchi co‐conceptualized the study, supervised the designing of the study protocol, provided guidance in the data analysis, reviewed the draft, and made important revisions to the manuscript. Both authors read and approved the final version of the manuscript for submission.

## Supporting information


**Appendix S1** Supporting Information.
